# Nuclear Transport Signals Control Cellular Localization and Function of Androgen Receptor Cofactor p44/WDR77

**DOI:** 10.1371/journal.pone.0022395

**Published:** 2011-07-15

**Authors:** Zhongping Gu, Liran Zhou, Shen Gao, Zhengxin Wang

**Affiliations:** 1 Department of Cancer Biology, The University of Texas MD Anderson Cancer Center, Houston, Texas, United States of America; 2 Department of Thoracic Surgery, Tangdu Hospital, Fourth Military Medical University, Xi'an, China; Chinese University of Hong Kong, Hong Kong

## Abstract

The androgen receptor (AR) cofactor p44/WDR77, which regulates expression of a set of androgen target genes, is required for differentiation of prostate epithelium. Aberrant localization of p44/WDR77 in the cytoplasm is associated with prostate tumorigenesis. Here, we describe studies that used the mouse prostate and human prostate cancer cells as model systems to investigate signals that control subcellular localization of p44/WDR77. We observed distinct subcellular location of p44/WDR77 during prostate development. p44/WDR77 localizes in the cytoplasm at the early stage of prostate development, when prostate epithelial cells are rapidly proliferating, and in the nucleus in adult prostate, when epithelial cells are fully differentiated. Subcellular localization assays designed to span the entire open-reading frame of p44/WDR77 protein revealed the presence of two nuclear exclusion signal (NES) and three nuclear localization signal (NLS) sequences in the p44/WDR77 protein. Site-directed mutagenesis of critical residues within an NLS led to loss of nuclear localization and transcriptional activity of p44/WDR77, suggesting that nuclear localization of p44/WDR77 is essential for its function as a transcriptional cofactor for AR. Three identified NLS were not functional in AR-positive prostate cancer (LNCaP and 22RV1) cells, which led to localization of p44/WDR77 in cytoplasm. The function of NLS in LNCaP cells could be restored by factor(s) from Cos 7 or PC3 cells. Mass spectrometric (MALDI-TOF/TOF) analysis identified proteins associated with an NLS and an NES in prostate cancer cells. These results provide a basis for understanding subcellular transport of p44/WDR77 during prostate development and tumorigenesis.

## Introduction

The p44/WD77 protein contains 342 amino acid residues and seven putative WD-40 repeats, interacts with androgen receptor (AR), and regulates expression of a set of androgen target genes in the prostate gland and in prostate cancer [1]–[4]. Prostate glands from *p44/WDR77*-deficient mice were smaller than those from wild-type mice, had fewer branches and terminal duct tips, and were deficient in production of secretory proteins [4]. In the *p44/WDR77*-null prostate, epithelial cells were not fully differentiated, and expression of androgen-regulated genes was altered. These results demonstrate that p44/WDR77 acts as an AR cofactor, playing important roles in prostate growth and differentiation by modulating AR target gene expression.

Immunohistochemical staining of prostate specimens showed that the p44/WDR77 protein localizes in the nucleus of benign epithelial cells and in the cytoplasm of prostate cancer cells [2]. Translocation of p44/WDR77 from the nucleus to the cytoplasm occurs in prostatic intraepithelial neoplasia and prostate cancer lesions [2], [3]. Forced nuclear localization of p44/WDR77 inhibited growth of prostate cancer cells in tissue culture [2] and completely abolished growth of prostate tumor xenografts in nude mice [3]. This growth inhibition was associated with upregulation of *p21* and *p27* gene expression; downregulation of *cyclin A, cyclin B*, and *CDK2* gene expression; and cell cycle arrest at the G_1_/G_0_ phase [2], [3]. Thus, p44/WDR77's function is regulated by its subcellular localization.

The protein sequence of p44/WDR77 is identical to that of a component (MEP50) of the methylosome complex [5] and a subunit (WD45) of the SMN complex [6]. The methylosome complex contains PRMT5, pICln, and Sm proteins and mediates assembly of spliceosomal snRNP [7]. SMN, the protein involved in spinal muscular atrophy, is part of a complex that contains the Sm protein and PRMT5. SMN complex is necessary and sufficient for assembly of UsnRNA [8], [9]. The methylosome and SMN complexes were isolated from cytoplasm of HeLa cells [7], [8], and the p44-containing complex was purified from a HeLa nuclear extract [1]. p44/WDR77 forms distinct complexes with various proteins, suggesting that it may have multiple roles.

Nuclear transport is proving to be a fundamental and critical mechanism for regulating protein localization and function. Deregulation of nuclear transport is implicated in the mislocalization and altered function of a variety of proteins [10]. The mistargeting of tumor suppressors can have dire cellular consequences that potentially lead to initiation and progression of cancer [11]. Protein transport in either direction across the nuclear envelope involves sequential steps, including (i) recognition of the protein import/export signal by an import/export receptor, (ii) docking of the protein/receptor assembly at the nuclear pore complex, (iii) release of the transported protein, and (iv) recycling of transport factors [12]. Although broadly defined, each of these steps is complex and involves intricate interplay of multiple protein components and a variety of recognition signals. Some proteins are not transported constitutively, but rather are imported or exported in response to signals, thus allowing their regulated redistribution within the cell. Transport recognition signals include the nuclear localization signal (NLS) and nuclear exclusion signal (NES). The basic NLS consists of a short stretch of positively charged lysine and arginine residues [13], [14]. The best-characterized NES is the leucine-rich NES; a protein containing this NES is exported by the export receptor CRM1 [15].

Here we report our observations of subcellular localization of p44/WDR77 during prostate development. We characterized the nuclear export and import signals in the p44/WDR77 protein and found that the p44/WDR77 molecule contains two NES and three NLS signals. The NLS signals did not function in AR-positive prostate cancer (LNCaP and 22RV1) cells, which might explain the localization of p44/WDR77 in the cytoplasm of these cells. Nuclear localization of p44 is essential for its function as a cofactor in AR-driven transcription. Our findings indicate that the mechanisms controlling p44/WDR77 subcellular localization and function are complicated.

## Results

### Distinct subcellular localization of p44/WDR77 protein during prostate development

**Figure 1:**
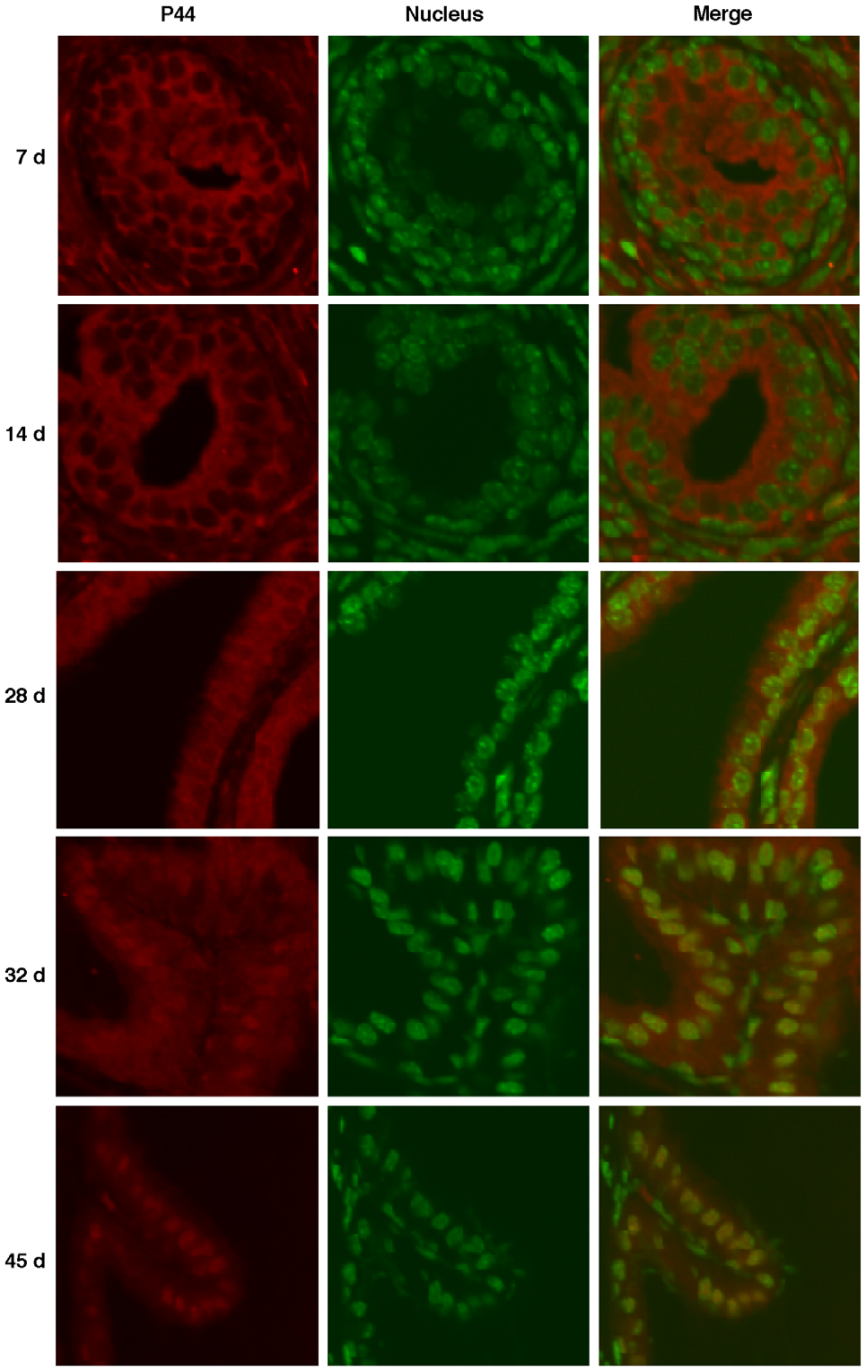
p44/WDR77 nuclear translocation during mouse prostate development. Immunostaining of p44/WDR77 (left panels, red) in prostate glands derived from mice aged 7, 14, 28, 32, or 45 days. The nucleus was counterstained with SYTOX Green (middle panels, green). The samples were observed under a confocal microscope.

The p44/WDR77 protein localizes in the nucleus of prostate epithelial cells [2], [4] and is transported into the cytoplasm in prostate intraepithelial hyperplasia and prostate cancer [2]. We investigated subcellular localization of p44/WDR77 in epithelial cells during mouse prostate development. Prostate glands were dissected from male mice at different ages and sections were immunostained with the anti-p44/WDR77 antibody (Figure 1, left panels, in red). The nucleus was counterstained with SYTOX Green (middle panels, in green). The p44/WDR77 protein localized in the cytoplasm of epithelial cells 7 days after birth and during the process of ductal branching morphogenesis (14–21 days after birth) [16]. Translocation of p44/WDR77 to the nucleus was first observed at age 28 days, and the nuclear accumulation peaked at age 45 days.

Antibodies to cytoskeletal keratin (CK) subclasses have been used to study prostate cell differentiation [17]. These studies demonstrated that changes in level of differentiation are accompanied by a distinct transition in the expression profiles of individual keratins. Epithelial cells in the adult prostate could be characterized as basal cells (CK5^+^/CK18^−^), luminal cells (CK5^−^/K18^+^), or intermediate cells (CK5^+^/K18^+^). The intermediate cells are in an intermediate state of differentiation and eventually differentiate into luminal cells [18]. Sections of prostate glands were stained with anti-keratin 5 (CK5) antibody. CK5 was detected in undifferentiated luminal cells (in the prostates of mice aged less than 28 days) but not in fully differentiated luminal cells (in the prostates of mice aged more than 32 days) (Figure S1A, middle panels). CK5 was strongly stained in all basal cells of prostates of mice aged more than 14 days. Staining of the proliferation marker Ki67 revealed that a majority (70%) of prostate epithelial cells were proliferative in mice aged 7 days and that the proliferation rate decreased dramatically in mice aged 14 to 32 days (Figure S1A, bottom panels; Figure S1B). Epithelial cell proliferation decreased even further in the prostate of mice aged 45 days. Thus, p44/WDR77 nuclear importation is associated with decrease of proliferation and functional cytodifferentiation of epithelial cells, coinciding with expression of prostate-specific secretory proteins [19]–[22]. p44/WDR77 nuclear staining remained strong in epithelial cells of adult prostate. Similarly, p44/WDR77 localized in the cytoplasm of epithelial cells in the human fetal prostate gland and in the nucleus in the adult prostate gland (P. Lee and Z. Wang, unpublished observations). Thus, transport of p44/WDR77 from the cytoplasm to the nucleus occurs during prostate development. In contrast, subcellular localization of AR did not change during mouse prostate development (Figure S1A, top panels).

### Subcellular localization of p44/WDR77 in various cancer cell types

**Figure 2:**
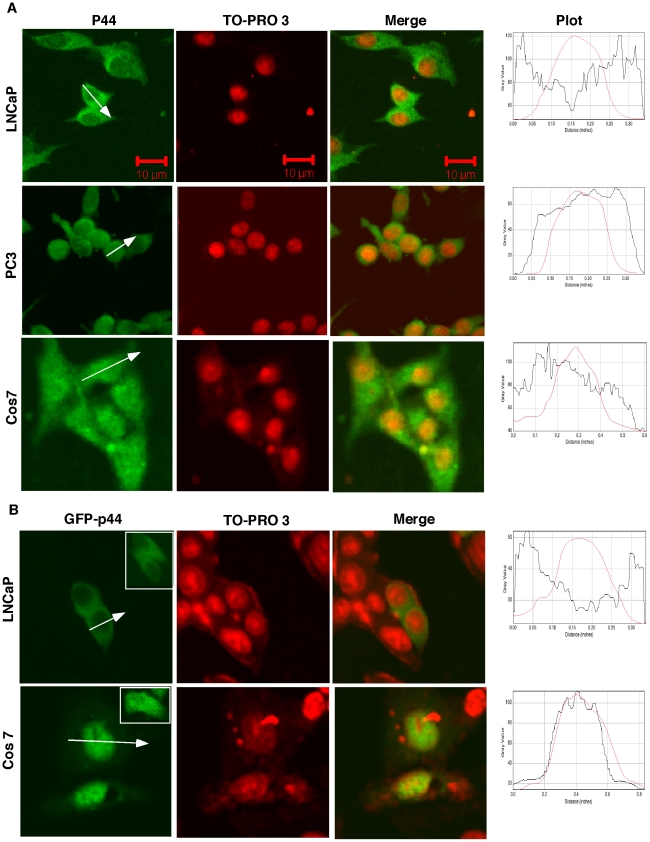
Subcellular localization of p44/WDR77 in cancer cell lines. (A) The p44/WDR77 protein was immunostained with the anti-p44/WDR77 antibody, and the cell nucleus was counterstained with TO-PRO 3. The samples were observed under a confocal microscope. (B) Cells were transfected with pcDNA-GFP-p44/WDR77 and observed under a confocal microscope. The cell nucleus was counterstained with TO-PRO 3. Inserts show cytoplasmic localization of GFP-p44/WDR77 in the stable LNCaP cell line expressing GFP-p44/WDR77 (top panel) and subcellular localization of GFP in Cos 7 cells (bottom panel). The fluorescence intensity changes across the white line in the direction of the arrowhead were plotted as the line intensities in the histogram (ImageJ, NIH) at the right side. The red line represents the intensities of nuclear staining.

Immunostaining and Western blot with the anti-p44/WDR77 antibody revealed that p44/WDR77 is cytoplasmic in AR-positive prostate cancer LNCaP (Figure 2A, top panels) and 22RV1 cells, in agreement with our previously reported data [2]. p44/WDR77 protein localizes in both nucleus and cytoplasm of AR-negative prostate cancer PC3 cells (Figure 2A, middle panels). Similarly, it is in both cytoplasm and nucleus of monkey fibroblast-like Cos 7 cells (Figure 2A, bottom panels), large T antigen−immortalized prostate epithelial BPH-1 cells, and other nonprostate cancer cells (Hela, 293T, and U87) (data not shown).

The *N*-terminal enhanced green fluorescent protein (GFP)-tagged p44/WDR77 (GFP-p44) was transiently expressed in LNCaP cells and, as shown in Figure 2B (top panels), the resulting GFP-p44 fusion protein had a subcellular localization in those cells similar to that of the endogenous p44/WDR77 protein (Figure 2A, top panels). An LNCaP cell line that stably expressed GFP-p44 was established, and the GFP-p44 fusion protein also localized in the cytoplasm of those cells (Figure 2B, top panel, insert). In contrast, GFP-p44 accumulated more in the nucleus of the Cos 7 cells (Figure 2B, bottom panels). Control GFP localized in both nucleus and cytoplasm in Cos 7 (Figure 2B, bottom panel, insert) and LNCaP cells [2]. Western blot of cytoplasmic and nuclear fractions further confirmed p44/WDR77 subcellular localization in these cells (Figure S2).

p44/WDR77 physically interacts with AR (Figure S3) and regulates AR-driven gene expression [1], [2], [4]. PC3 and Cos 7 cells do not express AR, but LNCaP and 22RV1 cells do (Figure S4A). We investigated whether AR expression influences subcellular localization of p44/WDR77. We established a stable PC3 cell line expressing AR (PC3-AR) (Figure S4A, lane 5), but expression of AR did not affect subcellular localization of p44/WDR77 in these cells (Figure S5). Similar results were obtained with Cos 7 cells (data not shown). These results indicate that AR does not directly involve in subcellular distribution of p44/WDR77. Furthermore, subcellular localization of p44/WDR77 in LNCaP and PC3-AR cells was not affected by the presence or absence of an androgen in the growth medium (Figure S5). In contrast, ligand-dependent translocation of AR was observed in these cells (Figure S4B). For reasons that are not clear, the p44/WDR77 physically interacts with AR but does not transport together with AR during androgen stimulation. It is more likely that AR in the cytoplasm associates with molecular chaperons and this interaction prevents its interaction with p44/WDR77.

### p44/WDR77 includes two functional NES

To identify the molecular determinants for subcellular localization of p44/WDR77, overlapping fragments spanning the entire open-reading frame of p44/WDR77 were cloned in frame to generate pcDNA-f:GFP-p44 fusion constructs. These constructs were transfected into Cos 7 cells to determine the critical regions of p44/WDR77 necessary for nuclear export or import.

**Figure 3:**
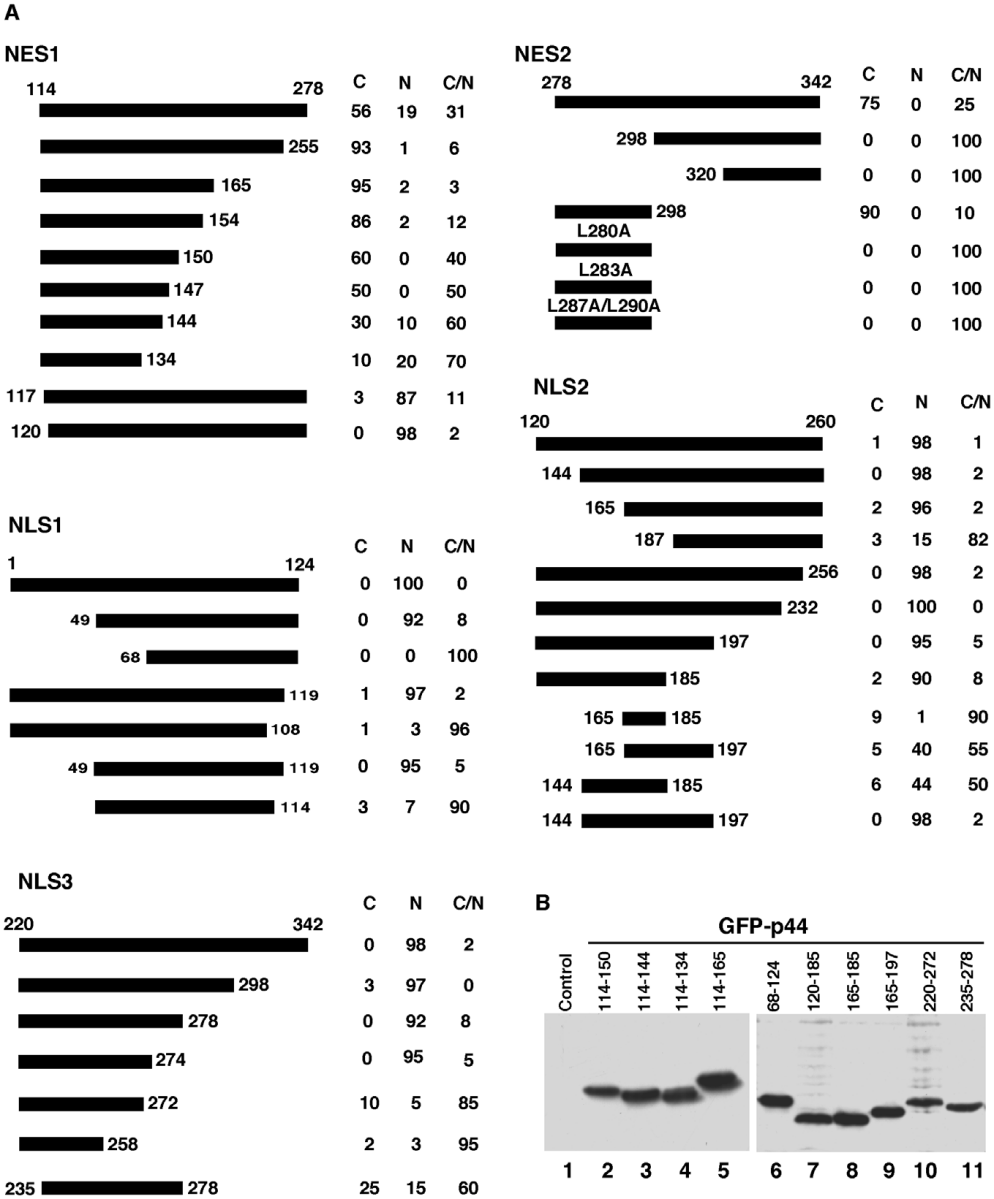
Mapping of nuclear transport signals in the p44/WDR77 molecule. (A) Diagrams of the p44/WDR77 truncations expressed as GFP-fusion proteins in Cos 7 cells. The percentages of cells with GFP-p44/WDR77 truncations in cytoplasm (C), nucleus (N), or cytoplasm plus nucleus (C/N) are shown on the right. (B) Western blot analysis of whole-cell extracts derived from Cos 7 cells transfected with pcDNA-f:GFP-p44/WDR77 truncations (lanes 2–11) with anti-FLAG antibody. Lane 1 is the whole-cell lysate from nontransfected Cos 7 cells.

**Figure 4:**
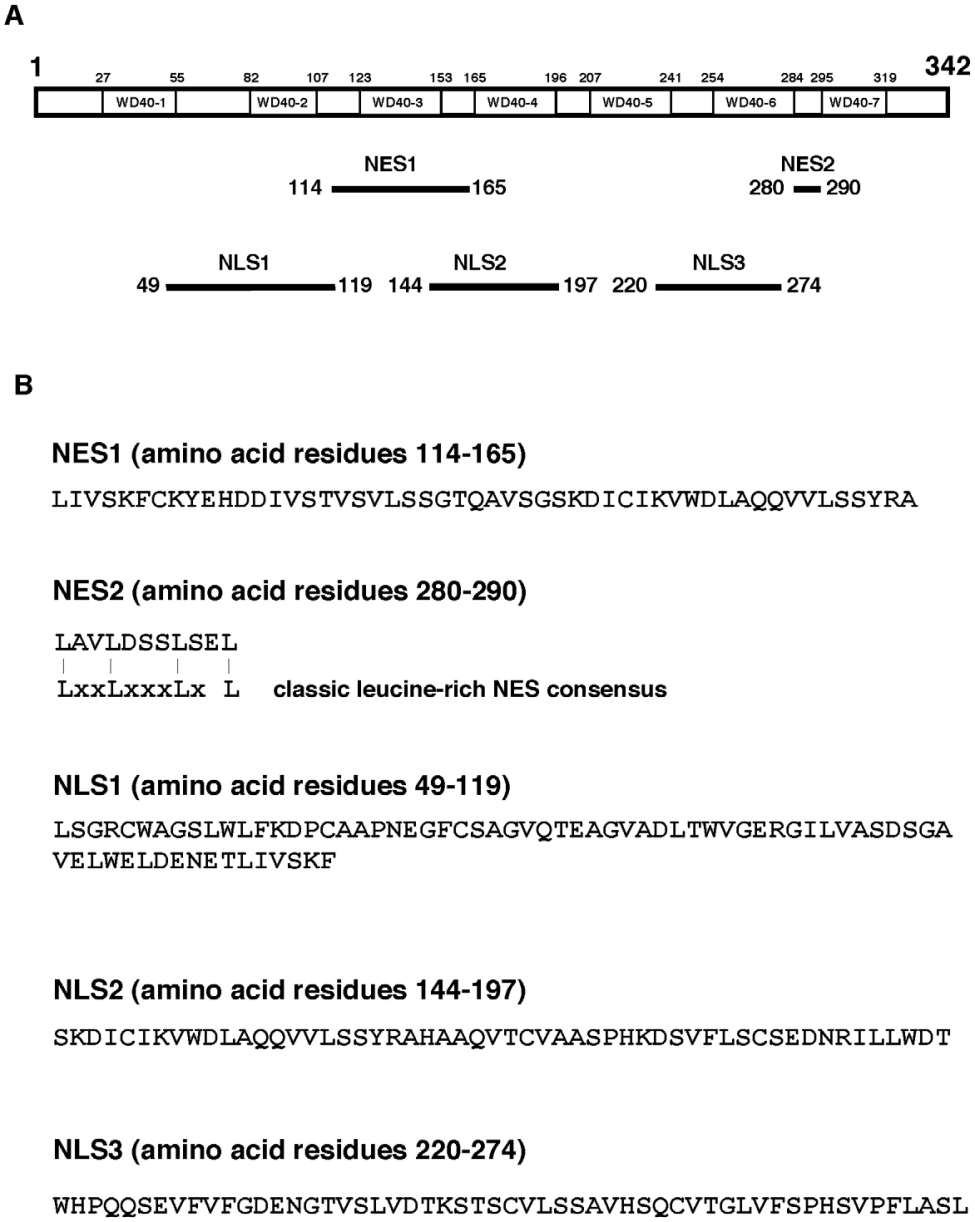
(A) Diagram shows the identified NES (NES1, NES2) and NLS (NLS1, NLS2, NLS3) in human p44/WDR77 protein. The seven WD40 repeats are indicated. (B) Amino acid sequences of identified nuclear transport signals and sequence alignment of NES2 with the consensus of the leucine-rich NES.

Two protein fragments, p44(114–278) and p44(278–342), were found within the cytoplasm in 56% and 75% of transfected cells, respectively (Figure 3A), suggesting that these fragments contain elements required for localization in cytoplasm. Deletion of 23 amino acid residues from the *C*-terminal end of the p44(114–278) fragment enhanced its cytoplasmic localization, indicating that this region contains an element (NLS3, below) that promotes nuclear localization. Further deletions from the *C*-terminal led to gradual loss of cytoplasmic localization. In contrast, deletion of three amino acid residues (^114^LIV^116^) from the *N*-terminal end of the p44(114–278) fragment abolished cytoplasmic localization, indicating that these three amino acid residues are critical for cytoplasmic localization of this fragment. The mutation (L114R/I115W/V116R) abolished nuclear export of p44(114–278). The region p44(114–165) was found within the cytoplasm in 95% of transfected cells (Figure 3A) and is a novel NES, designated NES1 (Figure 4B). NES1 does not resemble the conventional leucine-rich NES [15].

Deletion of 20 or 58 amino acid residues from the *N*-terminal end of the fragment p44(278–342) completely abolished its cytoplasmic localization (Figure 3A), whereas deletion of 44 amino acids from the *C*-terminal end did not decrease cytoplasmic localization. The region spanning amino acid residues 278 to 298 localized to the cytoplasm in 90% of transfected cells. Thus, the p44(278–298) fragment is a functional NES, designated NES2. Indeed, sequence analysis indicated that there is a leucine-rich sequence in this region (Figure 4B) that resembles the classical leucine-rich NES [15]. As expected, mutations on L280 or/and L283 abolished NES2 activity (Figure 3A). Thus, NES2 is a conventional leucine-rich NES.

### Three functional NLS found in p44/WDR77

Sequence deletion analysis indicated that three regions (amino acid residues 1–124, 120–260, and 220–342) contain NLS signals (Figure 3A). Deletion of amino acid residues 1 to 48 from the *N*-terminal end or of five amino acid residues (120 to 124) from the *C*-terminal end of the p44(1–124) fragment did not affect the NLS activity. Further deletion from either end resulted in complete loss of nuclear localization. The protein fragment p44(49–119) was found within the nucleus in 95% of transfected cells. Thus, the 49 to 119 region has nuclear targeting activity and an NLS (NLS1) was identified that does not resemble any known NLS (Figure 4B).

Deletion to amino acid residue 165 from the *N*-terminal end or to amino acid residue 185 from the *C*-terminal end of p44(120–260) did not affect nuclear localization (Figure 3A). However, the region from 165 to 185 does not have any detectable NLS activity. Inclusion of 21 amino acid residues (144 to 164) at the *N*-terminal end or 12 amino acid residues (186 to 197) at the *C*-terminal end partially restored NLS activity. The protein fragment p44(144–197) was found within the nucleus in 98% of transfected cells. Thus, NLS2 spans the region of amino acid residues 144 to 197 and is a novel NLS (Figure 4B).

Similar analysis defined a third NLS, NLS3, which contains amino acid residues 220 to 274 (Figure 3A). Amino acid residues ^272^SL^274^ are critical for nuclear targeting of p44(220–274), since deletion or mutation (^272^SL^274^ to ^272^GG^274^) of two amino acid residues led to complete loss of nuclear localization. This NLS is novel and does not resemble any known NLS (Figure 4B).

**Figure 5:**
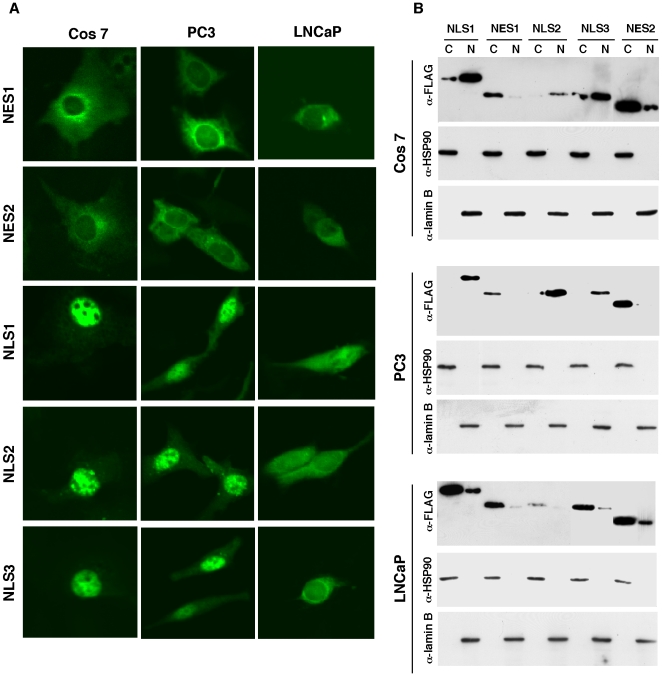
Subcellular localization of isolated nuclear export and import signals in Cos 7, PC3, and LNCaP cells. (A) Cells were transfected with pcDNA-f:GFP-NES1, -NES2, -NLS1, -NLS2, or -NLS3 and observed under a confocal microscope. (B) Western blot of cytoplasmic and nuclear fractions of the transfected cells described in (A) with anti-FLAG, anti-HSP90, or anti-lamin B antibody.

Western blot analysis with anti-FLAG antibody was used to detect expression and stability of all f:GFP-p44 truncations in Cos 7 cells (ten f:GFP-p44 truncations are shown in Figure 3B). The positions in the p44/WDR77 molecule and sequences of these identified nuclear export and import signals are shown in Figures 4A and 4B, respectively. The subcellular localization of identified nuclear transport signals in Cos 7 cells is shown in Figure 5.

### Mechanisms that control subcellular localization of p44/WDR77

**Figure 6:**
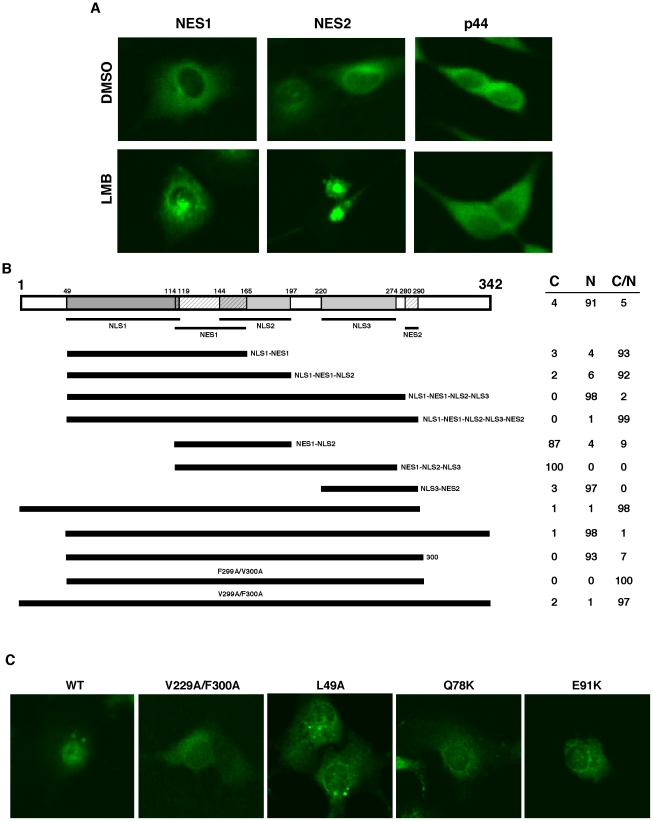
The relationship between identified nuclear export and import signals. (A) LMB does not inhibit translocation of p44/WDR77 to the cytoplasm. Cos 7 cells were transfected with pcDNA-GFP-NES1 or -NES2 (left two panels). The transfected cells were grown in the presence of dimethylsulfoxide (DMSO) or 20 mM of LMB for 6 hr. Subcellular localization of GFP-NES1 and GSP-NES2 was observed under a confocal microscope. Cos 7 cells were grown in the presence of DMSO or 20 µM of LMB for 6 hr and immunostained with the anti-p44 antibody (right panel). (B) Cos 7 cells were transfected with constructs expressing GFP-fusion proteins of various p44/WDR77 truncations or point mutations as indicated. The transfected cells were stained with TO-PRO 3 and observed under a confocal microscope. The percentages of cells with GFP signals in cytoplasm (C), nucleus (N), or cytoplasm plus nucleus (C/N) are shown on the right. (C) Point mutations on p44/WDR77 abolished its nuclear localization in Cos 7 cells. Cells were transfected with constructs expressing the wild-type or mutant (V299A/F300A, L49A, Q78K, or E91K) GFP-p44/WDR77, stained with TO-PRO 3, and observed under a confocal microscope.

Multiple nuclear transport signals within a protein may act through complicated (cooperating or antagonizing) mechanisms to determine its intracellular localization. Treatment of transfected cells with leptomycin B (LMB), a compound that specifically inhibits the leucine-rich NES by blocking CAM1 activity and results in accumulation of NES-containing proteins in the nucleus [15], blocked cytoplasmic localization of NES2 (Figure 6A, middle panels), as expected, but did not affect the activity of NES1 (Figure 5A, left panels). LMB treatment did not affect cytoplasmic localization of p44/WDR77 in LNCaP cells (Figure 6A, right panels), suggesting that NES1 is sufficient to determine cytoplasmic localization of p44/WDR77. Moreover, the mutation (L287A/L290A) that abolished NES2 activity did not affect the cytoplasmic localization of p44/WDR77 in LNCaP cells (data not shown).

To evaluate the combinatorial effect of the identified nuclear transport signals, a series of p44/WDR77 protein fragments containing various numbers of NES, NLS, or both NES and NLS were generated (Figure 6B). The fragment p44(49–165), which contains NLS1 and NES1, was found in both nucleus and cytoplasm in 93% of transfected Cos 7 cells, indicating that these two signals antagonize each other. Addition of NLS2 to this fragment did not change its subcellular localization, but the region p44(49–274), which contains NLS1, NES1, NLS2, and NLS3, showed exclusive nuclear localization in 98% of transfected Cos 7 cells, suggesting that these three NLS signals act cooperatively to overcome NES1 activity. The region p44(114–197), containing NES1 and NLS2, and the region p44(114–278), containing NES1 and NLS2 and NLS3, showed cytoplasmic localization in 87% or 100% of cells, respectively, indicating that NES1 is dominant over NLS2 and NLS3. In contrast, the region p44(220–298), containing NLS3 and NES2, localized in the nucleus in 97% of cells, suggesting that NLS3 is dominant over NES2. The fragment p44(49–298) contains all identified nuclear transport signals (NES1, NES2, NLS1, NLS2, NLS3) and showed both nuclear and cytoplasmic localization in 99% of transfected cells. Inclusion of the *N*-terminal 48 amino acid residues (1–48) did not affect subcellular localization, but the fragments p44(49–342) and p44(49–300) localized in the nucleus in more than 90% cells, a proportion similar to that achieved by the full-length p44/WDR77 protein. This observation indicates that the amino acids residues ^299^FV^300^ play a critical role in determining the nuclear localization of the fragment p44(49–300). Mutation of ^299^FV^300^ to ^299^AA^300^ completely abolished nuclear localization of p44(49–300) and full-length p44 (Figures 6B,C).

We generated a panel of point mutations within the 49–119 region of NLS1 and found that three residue mutations (L49A, Q78K, and E91K) would abolish NLS1 activity and result in cytoplasmic localization of p44/WDR77 in Cos7 cells (Figure 6C).

### Nuclear localization is required for p44/WDR77 function in AR-driven transcription

**Figure 7:**
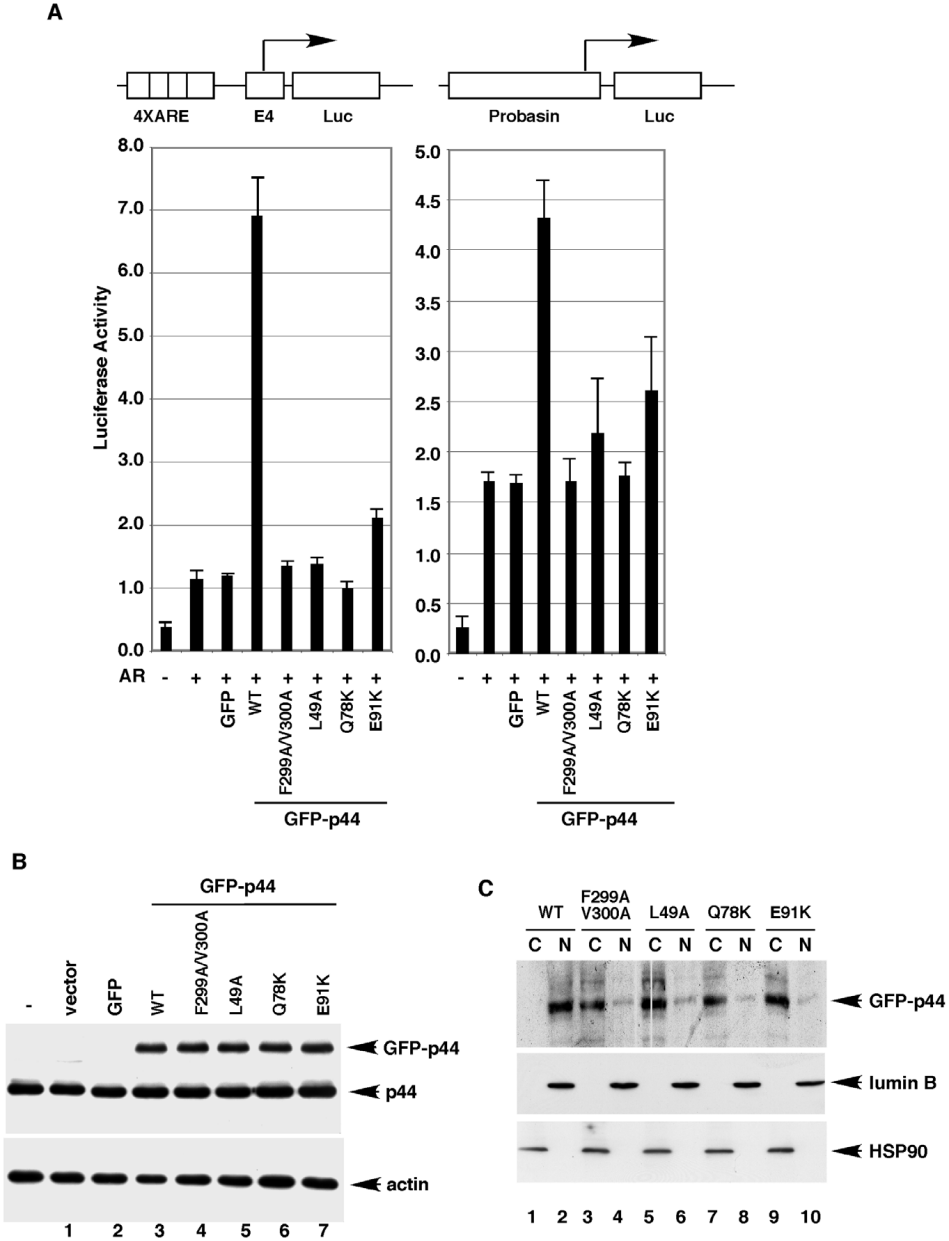
Nuclear localization is essential for p44/WDR77 function as an AR cofactor. (A) Cos 7 cells were transfected with 0.375 (left panel) or 0.75 (right panel) fmoles of pcDNA-AR, 25 fmoles of the report plasmid pGL3-4xARE-E4-luc (left panel) or pGL3-Probasin-luc (right panel), or 30 fmoles of pcDNA-f:GFP-p44 or pcDNA-f:GFP-p44mt. Transfected cells were grown in the presence of 10 nM R1881 for 48 hr and then harvested for luciferase assay. Values represent mean ± SD (n = 3). (B) Western blot analysis of whole-cell lysates (10 µg proteins per sample) derived from the transfected cells with the anti-p44 (top) or anti-actin (bottom) antibody as indicated. The transfected GFP-p44 fusion protein, endogenous p44 protein, and β-actin are indicated by arrows on the right. (C) Western blot of cytoplasmic and nuclear fractions of the transfected cells described in (A) with anti-FLAG, -HSP90, or anti-lumin B antibody.

Since p44/WDR77 enhances AR-driven gene transcription [1], the effect of mutations (F299A/V300A, L49A, Q78A, or E91K) that abolished nuclear localization of p44/WDR77 on AR-dependent transcription was investigated using a transient transfection assay. We co-transfected the luciferase reporter plasmid containing four tandem copies of the androgen-response elements (AREs) derived from the *PSA* gene upstream of the minimal adenovirus E4 promoter [23]–[25] with expression vectors for AR and wild-type or mutant GFP-p44/WDR77 fusion proteins into Cos 7 cells in the presence of the synthetic androgen R1881. As shown in Figure 7A (left panel), AR activated the reporter about 3-fold, and co-expression of wild-type GFP-p44 enhanced (∼6-fold) this AR-dependent transactivation. This result was consistent with our previous findings [1]. In contrast, co-expression of mutant (F299A/V300A, L49A, Q78A, or E91K) GFP-p44/WDR77 failed to increase this reporter activity. Similar results were obtained with a natural (Probasin) AR reporter (Figure 7A, right panel). Western blot analysis indicated that the wild-type and mutant GFP-p44/WDR77 proteins were expressed at similar levels (Figure 7B). Endogenous p44/WDR77 and β-actin were used as loading controls. Western blot analysis of cytoplasmic and nuclear fractions of transfected cells revealed that wild-type GFP-p44/WDR77 localized mainly in the nucleus and that mutation (F299A/V300A, L49A, Q78A, or E91K) of p44/WDR77 abolished its nuclear localization (Figure 7C). Thus, nuclear localization is required for the p44/WDR77 function in AR-driven transcription.

### The identified nuclear localization signals do not function in AR-positive prostate cancer cells

To test whether the identified nuclear transport signals function in prostate cancer cell lines (LNCaP, PC3, 22RV1), we performed localization assays using constructs expressing GFP-fusion proteins of NES1, NES2, NLS1, NLS2, and NLS3. All nuclear transport signals functioned similarly in AR-negative PC3 (Figure 5A, middle panels) and Cos 7 cells (Figure 5A, left panels). GFP-NES1 and GFP-NES2 were localized in the cytoplasm in LNCaP (Figure 5A, right panels) and 22RV1 (data not shown) cells. However, the GFP-fusion proteins of NLS1, NLS2, and NLS3 failed to localize in the nucleus of these cells (Figure 5A, right panels), indicating that these nuclear localization signals lost function in AR-positive LNCaP and 22RV1 cells. Western blots of cytoplasmic and nuclear fractions further confirmed the subcellular localization of identified nuclear transport signals in Cos 7, PC3, and LNCaP cells (Figure 5B).

**Figure 8:**
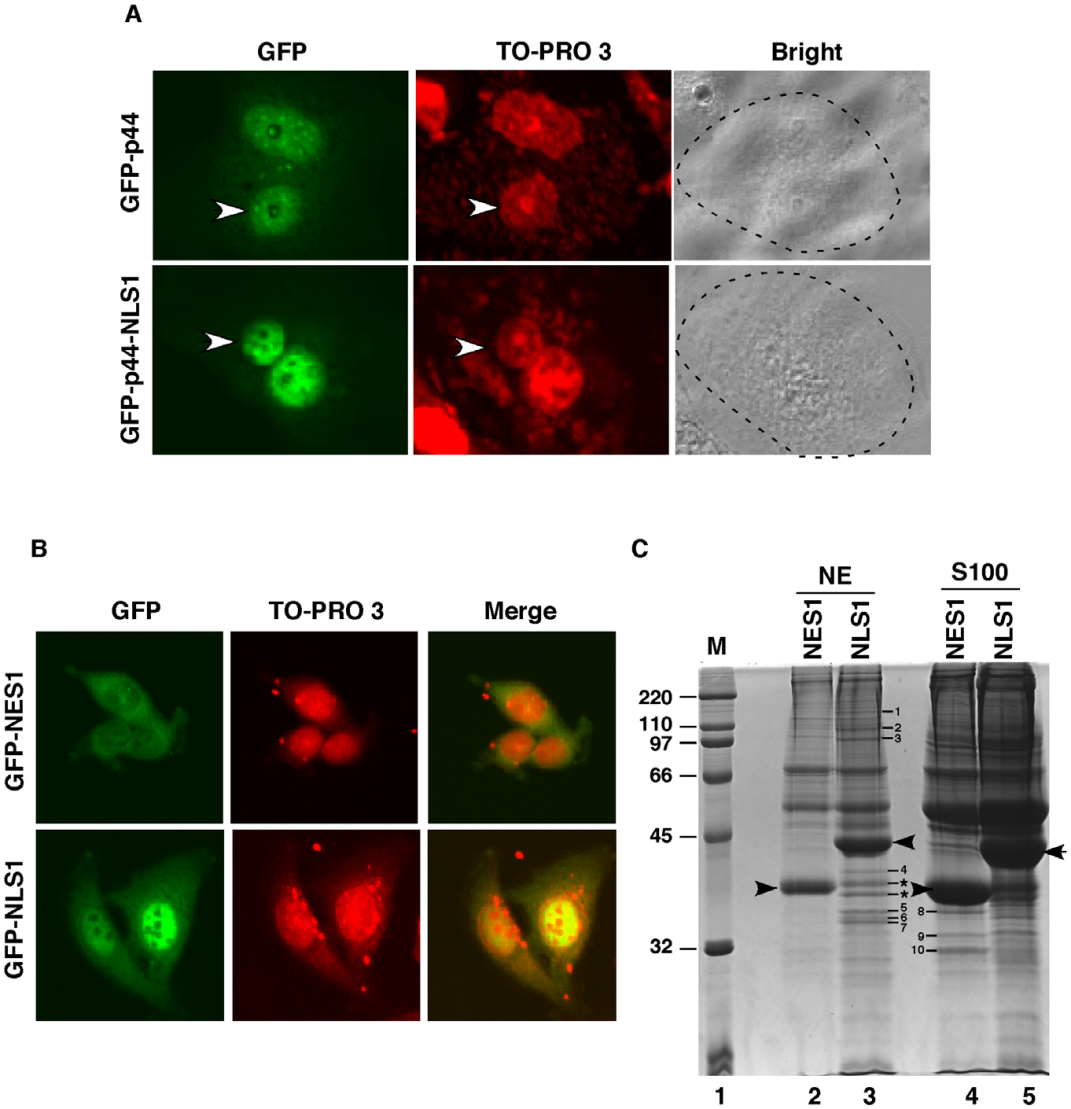
Cos 7 cells restore p44/WDR77 nuclear localization in LNCaP cells. (A) LNCAP cells were transfected with pcDNA-GFP-p44/WDR77 or pcDNA-GFP-NLS1. After expression of the transfected DNAs, the cells were fused to Cos 7 cells to form heterokaryons. After incubation for 6 hr, the cells were fixed and counterstained with TO-PRO 3, which differentiates the human and monkey nuclei within the heterokaryon (arrows identify the human nuclei). The panel marked Bright shows the phase-contrast image of the heterokaryons; the broken line highlights the cytoplasmic edge. (B) Cytoplasmic localization of f:GFP-NES1 and nuclear localization of f:GFP-NLS1 in PC3 cells. PC3 cells stably expressing f:GFP-NES1 (top panels) or f:GFP-NLS1 (bottom panels) were stained with TO-PRO 3 and observed under a confocal microscope. (C) Identification of polypeptides that were associated with NES1 or NLS1. The bands corresponding to f:GFP-NES1 and f:GFP-NLS1 are indicated by arrows. Polypeptides specifically associated with f:GFP-NLS1 or f:GFP-NES are indicated by numbers and stars. The standard protein markers (Bio-Rad) are shown in lane 1.

We then performed interspecies heterokaryon assays [26], [27] to investigate whether LNCaP cells express a factor that inhibits the function of nuclear localization signals or is lacking a factor that is essential for the function of these signals. Cos 7 cells were transiently transfected with pcDNA-GFP-p44 or pcDNA-GFP-NLS1 (Figure 8A). Twenty-four hours after transfection, untransfected human LNCaP cells were co-cultured and fused with the transfected Cos 7 cells to form heterokaryons. To distinguish between human LNCaP and monkey Cos 7 nuclei, the cells were stained with TO-PRO 3. Six hours after cell fusion, GFP-p44 and GFP-NLS1 localized in both human (Figure 8A, small nuclei indicated by white arrows) and monkey nuclei. This indicates that LNCaP cells are lacking the factor or signal that is required for function of p44/WDR77 nuclear localization signals. To confirm this finding, we performed the *in vitro* transportation assay (Figure S6). Whole-cell extracts were made from PC3 or LNCaP cells and added to cultured LNCaP or PC3 cells. The whole-cell lysate derived from LNCaP cells did not affect the nuclear localization of the full-length p44/WDR77 protein in PC3 cells, whereas the whole-cell lysate derived from PC3 cells switched the localization of p44/WDR77 from cytoplasm to nucleus in LNCaP cells. As a negative control, the PC3-derived whole-cell lysate did not affect subcellular localization of GFP in LNCaP cells. These analyses confirmed that LNCaP cells lack the factor(s) that is required for the function of the identified nuclear localization signals.

### Polypeptides associated with NLS1 and NES1 in prostate cancer cells

To identify proteins that are involved in nuclear transport of NLS1 and NES1, we established three stable PC3 cell lines that express the FLAG epitope-tagged GFP-NLS1 (f:GFP-NLS1), or GFP-NES1 (f:GFP-NES1). f:GFP-NES1 mainly localized in the cytoplasm (Figure 8B, top panels), while f:GFP-NLS1 was found largely in the nucleus (bottom panels) in the stable PC3 cell lines. Large amounts (50 ml for each cell line) of cytoplasm (S100) and nuclear (NE) extracts were prepared from f:GFP-NLS1 and f:GFP-NES1 cell lines and were submitted for immunoprecipitation using M2 agarose. After elution with the FLAG peptide, the immunopurified proteins were analyzed by SDS-PAGE and stained with Coomassie Brilliant Blue R-250 (Figure 8C; bands corresponding to f:GFP-NES1 and f:GFP-NLS1 are indicated by black arrows [lanes 2–5]). Polypeptides that were specifically associated with f:GFP-NLS (lane 3) but not with f:GFP-NES1 (lane 2) in the nucleus and those that were associated with f:GFP-NES1 (lane 4) in the nucleus but not with NLS1 (lane 5) in the cytoplasm were marked by numbers (1-10) and stars and submitted for mass spectrometric (MALDI-TOF/TOF) analysis.

Two of the polypeptides (lane 3) are the degraded products of f:GFP-NLS1, and the other identified polypeptides are listed in Table 1. Eight polypeptides were specifically associated with f:NLS1 but not f:GFP-NES1 in the nucleus. DEAH box protein 9 (DHX9) and DEAD box protein 21 (DDX21) are RNA helicases that have been shown to function as transcriptional cofactors for BRCA1-, CREBBP-, NF-κB-, and JUN-activated transcription [28]–[31]. Association of these two proteins with f:GFP-NLS1 in the nucleus indicates that they may be involved in p44-mediated transcription. In the developing *Xenopus* embryo, nucleolin (NCL) was localized in cytoplasm up to the midblastula stage, but thereafter accumulated in the nucleus [32]. Nucleolin can function as a carrier for the import and export of proteins across nuclear membrane [33], [34]. Nucleophosmin (NPM) colocalized with nucleolin and also shuttled between cytoplasm and nucleus [35]. Nucleolin bound to histone H1 and induced chromatin decondensaion [36], [37]. Histone H1 (H1.3, H1t, H1.4, H1.2) found in the f:GFP-NLS1 complex may associate with nucleolin. Three polypeptides (LDH A chain, tumor protein D54, and heat shock protein b-1) were specifically associated with f:GFP-NES1 but not with f:GFP-NLS1 in the cytoplasm. The function of tumor protein D54 (TP52L) is unknown. The mRNAs of DHX9, DDX21, NCL, NPM, and TP52L were detected in LNCaP and PC3 cells (Figure S7).

**Table 1:** Proteins associated with NLS1 or NES1 in PC3 cells.

Sample ID	Protein Name	Accession No.	Protein MW	Peptide Counts
1	DEAH box protein 9	Q08211.4	140	29
2	Nucleolin	P19338.3	105	14
3	DEAD box protein 21	Q9NR30.5	100	26
4	Nucleophosmin	P06748.2	40	8
5	Histone H1.3/H1t	P16402/P22492.4	35	9/5
6	Histone H1.4	P10412	33	12
7	Histone H1.2	P16403	32	15
8	LDH A chain	P00338.2	36	12
9	Tumor protein D54	O43399.2	32	6
10	Heat shock protein ß-1	P04792.2	30	8

## Discussion

The functional activity of p44/WDR77, an important regulator of AR functions during prostate gland development and prostate tumorigenesis [1]–[4], is controlled by its subcellular localization. In this study, we demonstrated that p44/WDR77 can localize in the nucleus or cytoplasm and defined two NES and three NLS sequences that are responsible for nuclear import and export of p44/WDR77. We further found that p44/WDR77 NLS signals are not functional in AR-positive prostate cancer cells, resulting in accumulation of p44/WDR77 in the cytoplasm of such cells. Importantly, regulating of p44/WDR77 cellular distribution is independent of AR.

### p44/WDR77 comprises multiple nuclear transport signals

By studying the ability of various p44/WDR77 segments that determine nuclear or cytoplasmic localization of fused GFP, we defined the minimum essential two NES and three NLS sequences in the p44/WDR77 protein. A canonical NES (NES2) was identified in the *C*-terminal part of p44/WDR77, and mutations on the conserved leucine residues in NES2 that are important for the CRM1-dependent export function abolished its nuclear export activity. NES2 is conserved among p44/WDR77 proteins across different species and influences cytoplasmic localization of the fragment p44(49–278) (containing NLS1, NES1, NLS2, and NLS3). Mutations of the critical leucine residues had no affect on the cytoplasmic localization of full-length p44/WDR77 in LNCaP cells, indicating that NES2 is not essential for nuclear export of the full-length protein. In support of this finding was the observation that LMB, which inhibited nuclear export of isolated NES2, did not affect cytoplasmic localization of p44/WDR77 in LNCaP cells. One novel NES (NES1) was defined as a 52-amino-acid fragment (amino acid residues 114–165) that partially overlapped NLS1 (amino acid residues 49–119). NES1 functions in a CRM1-independent manner and is sufficient to mediate cytoplasmic export of p44/WDR77 in LNCaP cells.

We defined three NLS sequences, which we have mapped to amino acid residues 49–119, 144–197, and 220–274. These NLS signals are novel and do not resemble classical NLS, PY-NLS, or other known NLS signals. Mutations (L49A, Q78K, or E91K) that disrupt nuclear import of NLS1 are sufficient to abolish nuclear localization of the full-length p44/WDR77 in Cos 7 cells, indicating that the nuclear localization of p44/WDR77 is determined mainly by NLS1.

### Mechanisms for p44/WDR77 transport between cytoplasm and nucleus

The mechanism for p44/WDR77 nuclear translocation is further elucidated by the fragments that contain the identified nuclear transport signals. The nuclear export of NES2 or nuclear import of NLS2 and NLS3 is weak when compared to that of NES1 or NLS1. Fragments containing NES1 and NLS2 or/and NLS3 (but not NLS1) accumulated in the cytoplasm, while fragments containing NLS1 and NES2 (but not NES1) accumulated in the nucleus. The fragment containing both NLS1 and NES1 distributed equally in cytoplasm and nucleus, indicating comparable nuclear transport activities.

The *C*-terminal region (299–342) does not contain any nuclear export or import activity when fused to GFP. Interestingly, residues 299-300 could change the cytoplasmic localization of regions p44(49–298) and p44(1–298) to the nucleus, and mutation (V299A/F300A) abolished this activity. These results suggest that amino acid residues V299 and F300 play a critical role in nuclear localization of p44/WDR77. Understanding the exact mechanism of how these residues regulate subcellular localization of p44/WDR77 will require further investigation. One possibility is that these residues somehow modulate the nuclear import functions of p44/WDR77 by masking or sequestering the NES1 signal.

### The subcellular localization of p44/WDR77affects its function

Our studies revealed distinct subcellular localization of p44/WDR77 during mouse prostate development. During the early stage of prostate development, p44/WDR77 localizes in the cytoplasm when epithelial cells are rapidly dividing. In the adult prostate, p44/WDR77 is resident in the nucleus of fully differentiated epithelial cells. These observations led to the idea that nuclear p44/WDR77 is required for differentiation of prostate epithelial cells. This may be especially important during early prostate development when the p44/WDR77 function in differentiation is not required. Consistent with this notion was our finding that loss of the *p44/WDR77* gene resulted in loss of differentiation of prostate epithelium [4]. It is also possible that cytoplasmic p44/WDR77 functions to control cell growth, based on its presence in complexes that are involved in splicing.

While p44/WDR77 is resident in the nucleus of benign prostate epithelial cells, it localizes to the cytoplasm in prostate cancer cells. This translocation event occurs in hyperplastic epithelial cells. Forced nuclear localization of p44/WDR77 inhibited proliferation of prostate cancer cells [2], [3]. These observations suggest that nuclear p44/WDR77 inhibits cell growth and is associated with differentiation while its translocation to cytoplasm relieves this inhibition. Nuclear import of isolated NLS signals is defective in AR-positive prostate cancer (LNCaP and 22RV1) cells, leading to sequestering of p44/WDR77 into cytoplasm and relieve p44/WDR77-mediated growth inhibition.

Taken together, the results of this study provide several new insights into the molecular mechanisms governing nuclear and cytoplasmic localization of p44/WDR77. These data also provide important tools for further investigation of the signaling pathways controlling subcellular localization of p44/WDR77 during prostate development and prostate tumorigenesis. Identification of proteins that are specifically associated with NLS1 and NES1 would allow us to further investigate how these proteins are involved in the control of subcellular localization of p44/WDR77. The nucleophosmin gene is one of the most frequent targets of mutation, deletion, and chromosome translocation associated with a variety of human cancers [35]. Alteration or loss of nucleophosmin function is suggested to cause unregulated cell growth and proliferation. Of particular interest would be investigation of whether LNCaP cells have altered nucleophosmin, which would explain the cytoplasmic localization of p44/WDR77.

## Materials and Methods

### Prostate gland preparation and immunostaining

Experiments in mice were conducted according to NIH guidelines and approved by the Institutional Animal Care and Use Committee (IACUC #01-02-00134) at The University of Texas MD Anderson Cancer Center. Male mice (B57/CL) aged 7, 14, 28, 32, and 45 days were killed and the prostate glands were freed from the other structures. The prostate glands were removed *en bloc* (including the seminal vesicles, urethra, and bladder) and fixed with 4% paraformaldehyde in phosphate-buffered saline solution (PBS) at 4°C overnight and embedded in paraffin. Sections (4 µm) were cut and mounted on Super-frost Plus adhesion slides (Fisher). Antigen-purified anti-p44/WDR77 antibody (1∶100) [1] was applied to the prostate tissue sections and incubated overnight. Alexa 594-labeled anti-rabbit immunoglobulin G antibody (1∶1,000) (DAKO) was used as the second antibody. The nucleus was stained with SYTOX Green. The fluorescent signals were observed under a confocal microscope with a red (to detect the p44/WDR77 protein) or green (to detect the nucleus) filter.

### DNA constructs and reagents

The p44/WDR77 gene fragments were amplified from the pcDNA-p44/WDR77 construct [1] using specific primer pairs with appropriate restriction sites. The DNA fragment encoding the GFP, derived from pEGFP-N1 (Clontech), was subcloned into pcDNA3.1 to generate the pcDNA-f:GFP construct. The cDNA encoding human p44/WDR77 or a p44/WDR77 truncation was subcloned into pcDNA-f:GFP to express the *N*-terminal f:GFP-fusion proteins of p44/WDR77 or p44/WDR77 truncations. Site-directed mutagenesis was performed according to the QuickChange site-directed mutagenesis kit protocol (Stratagene). All constructs were verified by restriction enzyme digestion and by DNA sequencing. Methyltrienolone (R-1881) and LMB were obtained from NEN and LC Laboratories, respectively.

### Immunostaining and transfection experiments

LNCaP, PC3, 22RV1, BPH1, and Cos 7 cell lines were obtained from American Type Culture Collection and maintained in RPMI 1640 medium with 10% fetal bovine serum. Cells were grown on chamber slides, fixed with cold methanol (−20°C) for 10 min, and immunostained with the anti-p44/WDR77 antibody. The Alexa-555-label second antibody (Molecular Probes) was used. After washing, cells were stained with TO-PRO 3 (10 µg/ml) (Molecular Probes), mounted in Histogel (Linaris), and analyzed directly by fluorescence confocal microscopy. Transfection into these cell lines was performed with the Lipofectamine 2000 reagent (Invitrogen) according to the manufacturer's instructions. The cells were fixed 24 hr after transfection with cold methanol for 10 min, stained with TO-PRO 3 for 30 min, and analyzed by fluorescence confocal microscopy with a green (to detect the GFP or GFP-p44 fusion proteins) or red (to detect the nucleus counter-stained with TO-PRO 3) filter. For treatment with LMB, cells were grown in the presence of dimethylsulfoxide (control) or LMB (20 µM) for 6 hr. Cells were fixed with cold methanol for 10 min, stained with TO-PRO 3, and submitted for analysis.

### Cytoplasmic and nuclear extract preparation

Cytoplasmic and nuclear fractions were prepared from cultured cells by using the Nuclear Extract Kit (Catalog #40010 & 40410, Active Motif). Briefly, cells were washed with ice-cold PBS and removed from the dishes by gentle scraping. Cells were pelleted by centrifugation, resuspended in 500 µl Hypotonic Buffer, and incubated for 15 min on ice. Twenty-five microliter of Detergent was added and the sample was subjected to vortexing for 10 s at the highest setting. The cell lysate was subjected to centrifugation for 30 s at 14,000×*g* at 4°C and the supernatant (cytoplasmic fraction) was transferred into a microcentrifuge tube and stored at -80°C until needed. The nuclear pellet was resuspended in 50 µl Complete Lysis Buffer subjected to vortexing for 10 s, incubated for 30 min on ice, and subjected to centrifugation for 10 min at 14,000×*g* at 4°C. The supernatant (nuclear fraction) was transferred into a microcentrifuge tube and stored at −80°C.

### Heterokaryon assay

Interspecies heterokaryons of human LNCaP cells and monkey Cos 7 cells were formed according to the previously described method [38], [39]. Briefly, Cos 7 cells were transiently transfected with plasmid DNA (pcDNA-f:GFP-p44/WDR77 or pcDNA-f:GFP-NLS1). Twenty-four hours after transfection, nontransfected human LNCaP cells were co-plated with the transfected Cos 7 cells at a 2∶1 ratio and co-cultured for 18 hr. The co-cultured cells were fused with PEG6000 at 23°C for 5 min. Fused cells were washed with PBS and incubated at 37°C for an additional 6 hr. The cells were fixed with cold methanol for 10 min and counterstained with TO-PRO 3 to distinguish between human LNCaP and monkey Cos 7 cell nuclei.

### *In vitro* transport assay

*In vitro* transport assay was performed as described previously [40]. Briefly, LNCaP or PC3 cells were transiently transfected with pcDNA-f:GFP or pcDNA-f:GFP-p44/WDR77. Eighteen hours after transfection, cells were subjected to permeabilization with digitonin (55 µg/ml) and incubated for 30 min at 23°C with bovine serum albumin (20 mg/ml) or with whole-cell lysate of LNCaP or PC3 cells in transport buffer [20 mM Hepes, pH 7.4, 110 mM potassium acetate, 5 mM NaCl, 2 mM MgCl_2_, 1 mM EGTA, 1 mM dithiothreitol, 1 mM ATP, 10 mM creatine phosphate, 4 units/ml phosphokinase, and protease inhibitor cocktail (Sigma-Aldrich)]. The cells were fixed with cold methanol for 10 min, counterstained with TO-PRO 3, and observed under a confocal microscope.

### Luciferase assay

The pGL3-4xARE-E4-luc luciferase report contains four tandem copies of AREs of the *PSA* gene, upstream of the minimal adenovirus E4 promoter and the pGL3-Probasin-luc reporter contains the Probasin promoter from −244 to +24 [41]–[45]. Cos 7 cells (1.6×10^4^ cells/well) were plated into 24-well plates and transfected 24 hr later with 25 fmoles of a luciferase reporter plasmid, 0.8 fmoles of a pRL-LUC internal control plasmid, and indicated amounts of expression plasmids. The total amount of DNA was adjusted to 75 fmoles using pcDNA3.1. The transfection was conducted using Lipofectamine (Invitrogen) in serum- and phenol red-free RPMI 1640 medium. After 6 hr of transfection, the medium was exchanged for phenol red-free RPMI 1640 medium plus 10% dextran/charcoal-stripped fetal bovine serum and R-1881 (10 nM). Cells were cultured for another 48 hr and harvested for analysis in a dual luciferase assay (Promega). Three independent experiments were performed for each transient transfection assay, and results are presented as mean ± SD (n = 3).

### Establishment of PC3 lines that stably expressed a FLAG-tagged GFP-NLS1 or GFP-NES1 and immunopurification of f:GFP-NLS1− and f:GFP-NES1−associated proteins

PC3 cells were transfected with pcDNA-f:GFP, pcDNA-f:GFP-NLS1, or pBabe-f:GFP-NES1 and further incubated at 37°C for 1–1.5 days before being split 1∶6 for G418 selection (0.5 mg/ml). The medium was changed every 3 or 4 days. Individual G418-resistant colonies, normally seen after 2 weeks, were expanded into cell lines and then characterized by western blotting using the anti-FLAG M2 monoclonal antibody (Sigma-Aldrich). The cell lines expressing FLAG-tagged GFP, GFP-NLS1, or GFP-NES1 were further expanded and analyzed. Nuclear and cytoplasmic extracts were prepared according to our standard methods [46] from 200 plates (150 mm) of cells and used to immunopurify the f:GFP-NLS1− or f:GFP-NES1−containing complexes. Typically, 1 ml of nuclear or cytoplasmic extract was mixed with 10 µl of M2 agarose (Sigma-Aldrich) and incubated for 3 hr at 4°C with rotation. After five washings in 1 ml of a buffer containing 20 mM HEPES (pH 7.9), 0.2 mM EDTA, 20% glycerol, 2 mM dithiothreitol, 300 mM KCl, and 0.1% NP40, the bound proteins were eluted from the M2 agarose by incubation at 4°C for 30 min with 10 µl of the same buffer plus 0.2 mg/ml of the FLAG peptide (Asp-Tyr-Lys-Asp-Asp-Asp-Asp-Lys). The purified proteins were precipitated with 10% trichloroacetic acid and loaded onto a 12% polyacrylamide gel for SDS-PAGE. Proteins were visualized by silver or Coomassie Brilliant Blue R-250 staining. Protein bands were cut and sent to the Mass Spectrometry Laboratory (MD Anderson Cancer Center) for MALDI-TOF/TOF analysis.

### Real-time polymerase chain reaction

Total RNAs were isolated from LNCaP and PC3 cells by the TRIzol Reagent and reverse-transcribed by using the Reaction Ready First Strand cDNA Synthesis Kit (SuperArray Bioscience Corp.). The resulting cDNA products were PCR-amplified (40 cycles of 30 s at 94°C; 20 s at 55°C; 30 s at 72°C) with the RT^2^ real–time SyBRgreen PCR master mix and the gene-specific primer sets for human DXH9, DDX21, NCL, NPM, TP52L, and β-actin (SuperArray Bioscience Corp.) by the SmartCycler II (Cepheid). The raw data procession and quantification were performed with the SmartCycler Software (Version 2.0C). The 2^-ΔΔCT^ method was used to determine the relative quantification of target gene expression. The relative mRNA expression  = 1,000× quantification of the gene/quantification of actin.

## Supporting Information

Figure S1 (A), Immunostaining of AR, CK5, and Ki67 in prostate glands derived from mice age 7, 14, 28, 32, 45 days. The alkaline phosphophase - labeled anti-rabbit immunoglobulin G antibody was used. (B), The percentage of Ki67-positive epithelial cells in prostate glands derived from mice age 7, 14, 28, 32, 5 days.
(TIF)

Figure S2 Western blot of cytoplasmic and nuclear fractions of Cos 7, LNCaP, and PC3 cells with anti -p44 (top), -HSP90 (middle), or -lamin B antibody.
(TIF)

Figure S3 P44 physically interacts with AR. GST (lanes 2, 8, and 13) or GST-p44 truncations, immobilized on beads, were mixed with 5 ml of in vitro labeled AR. After washing the beads, the bound proteins and 10% of the input (lanes 1, 7, 12) were analyzed on 8% SDS-PAGE and visualized by autoradiography.
(TIF)

Figure S4 (A), AR expression in prostate cancer cell lines. Western blot with anti-p44, -AR, and -actin antibodies were performed using whole-cell lysates made from Cos 7, LNCaP, PC3, and PC3-AR cells. (B), Ligand-dependent nuclear translocation of AR. LNCaP and PC3-AR cells were grown in the absence or presence of 10 nM R1881overnight and submittedfor AR immunostaining. The nucleus was stained with TO-PRO 3 and the fluorescent signals were observed under a confocal microscope.
(TIF)

Figure S5 Subcellular localization of p44 was not affected by the androgen. LNCaP and PC3-AR cells were grown in the absence or presence of 10 nM R1881overnight and submitted for p44 immunostaining. The nucleus was stained with TO-PRO 3 and the fluorescent signals were observed under a confocal microscope.
(TIF)

Figure S6 Restoration of p44/WDR77 nuclear localization in LNCaP cells by PC3 whole-cell lysate. LNCaP and PC3 cells were transfected with pcDNA-GFP-p44/WDR77 or pcDNA-GFP. After 24 hr, the cells were washed and incubated with whole-cell lysate derived from LNCaP or PC3 cells as indicated. The fluorescent signals were observed under a confocal microscope.
(TIF)

Figure S7 Expression of identified factors in PC3 and LNCaP cells. Total RNAs were isolated from LNCaP and PC3 cells and submitted for RT-PCR analysis with primers specific for identified factors.
(TIF)
