# Supplementary figures and images for: Nuclear Transport Signals Control Cellular Localization and Function of Androgen Receptor Cofactor p44/WDR77
Source: PLoS One. 2011 Jul 15;6(7):e22395. doi: 10.1371/journal.pone.0022395 (PMC3137635; doi:10.1371/journal.pone.0022395)

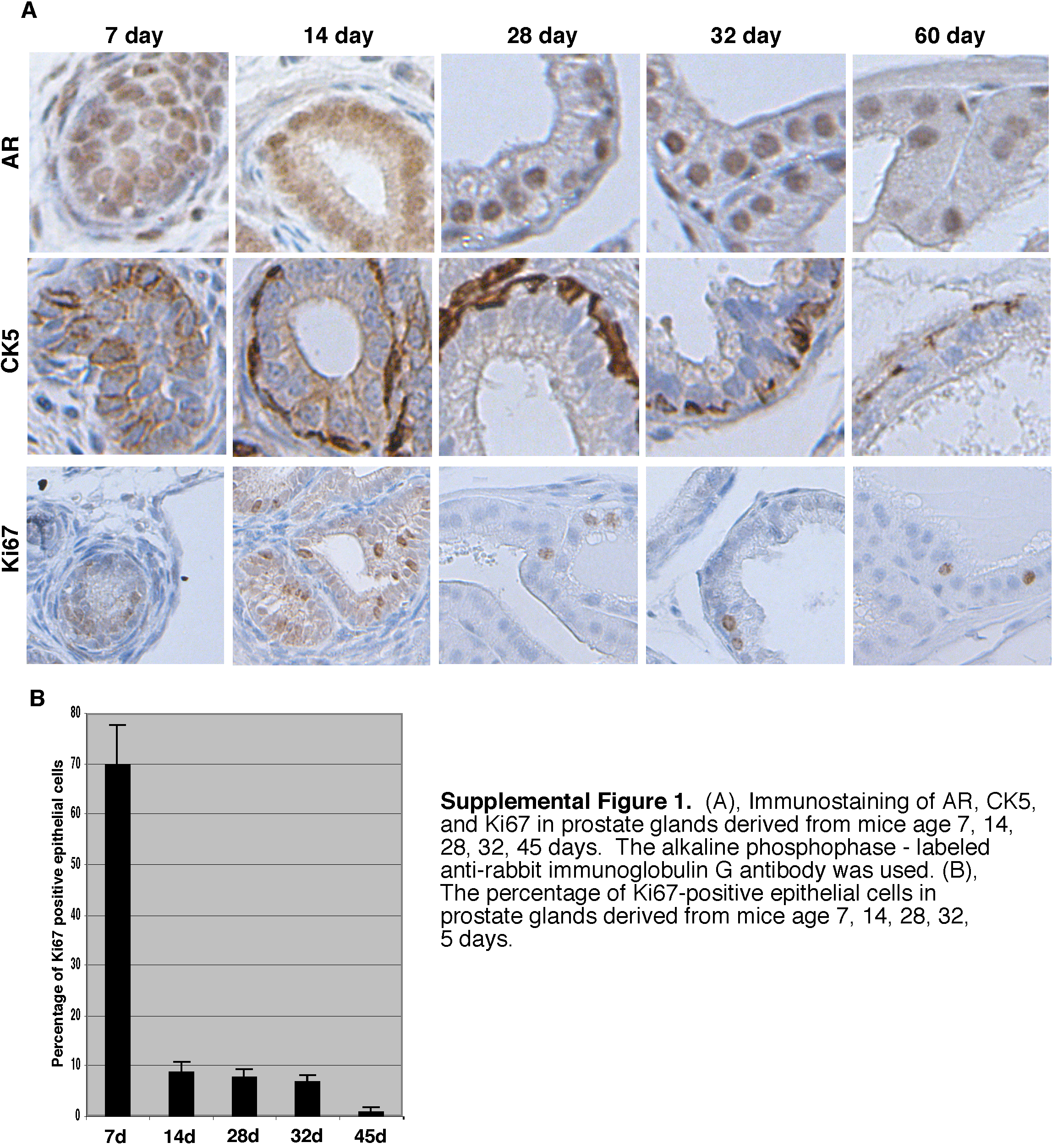

Supplement: Figure S1 — (A), Immunostaining of AR, CK5, and Ki67 in prostate glands derived from mice age 7, 14, 28, 32, 45 days. The alkaline phosphophase - labeled anti-rabbit immunoglobulin G antibody was used. (B), The percentage of Ki67-positive epithelial cells in prostate glands derived from mice age 7, 14, 28, 32, 5 days. (TIF) [file pone.0022395.s001.tif]

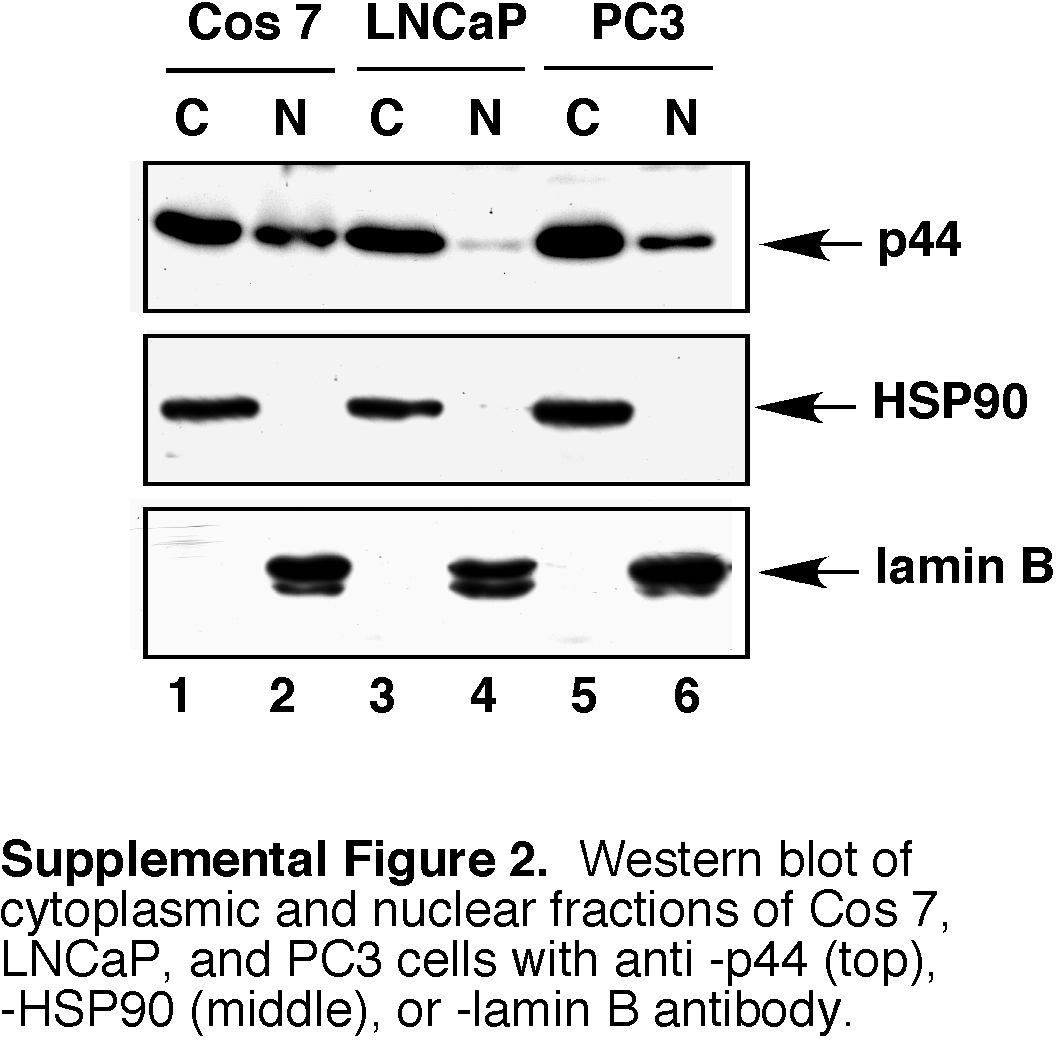

Supplement: Figure S2 — Western blot of cytoplasmic and nuclear fractions of Cos 7, LNCaP, and PC3 cells with anti -p44 (top), -HSP90 (middle), or -lamin B antibody. (TIF) [file pone.0022395.s002.tif]

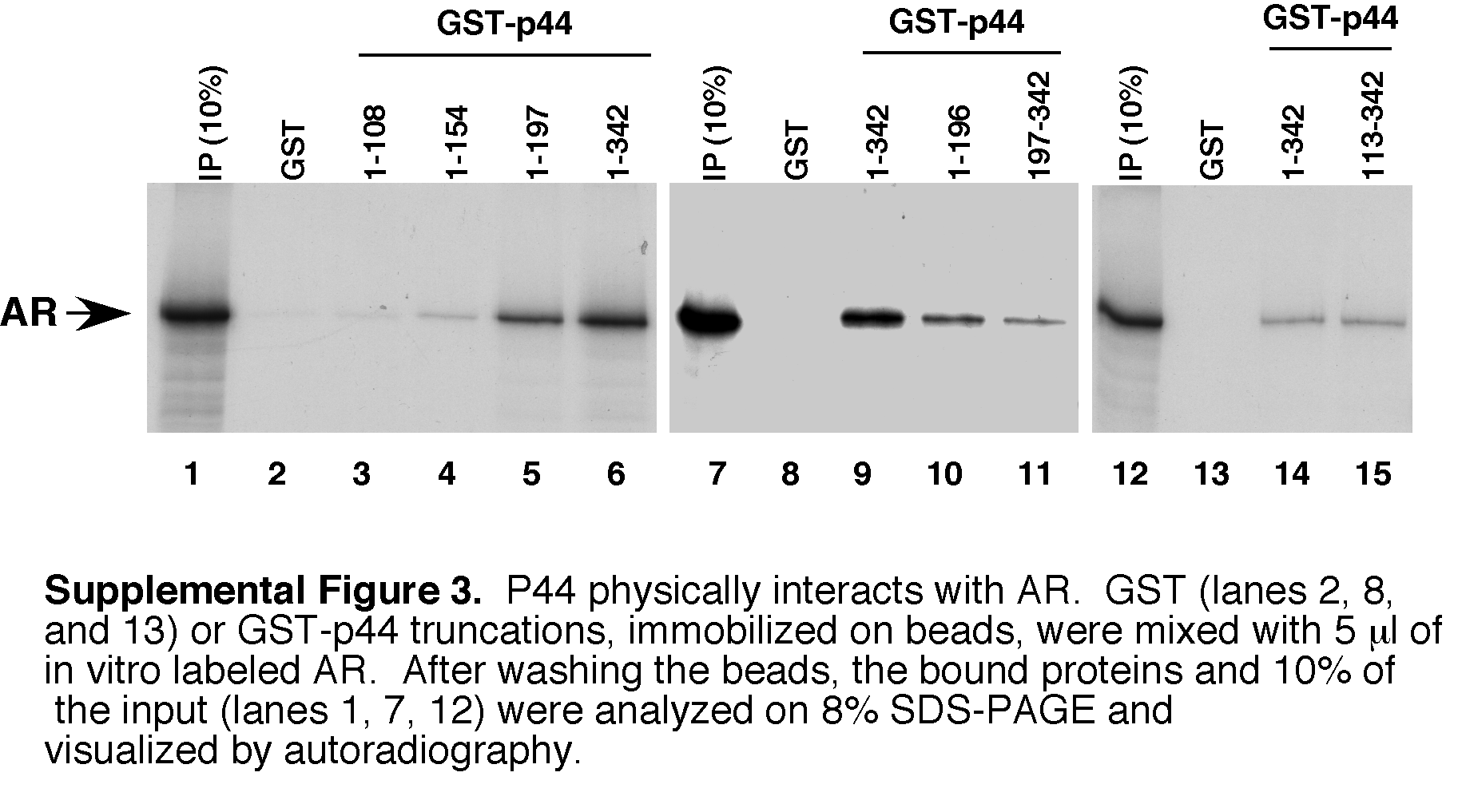

Supplement: Figure S3 — P44 physically interacts with AR. GST (lanes 2, 8, and 13) or GST-p44 truncations, immobilized on beads, were mixed with 5 ml of in vitro labeled AR. After washing the beads, the bound proteins and 10% of the input (lanes 1, 7, 12) were analyzed on 8% SDS-PAGE and visualized by autoradiography. (TIF) [file pone.0022395.s003.tif]

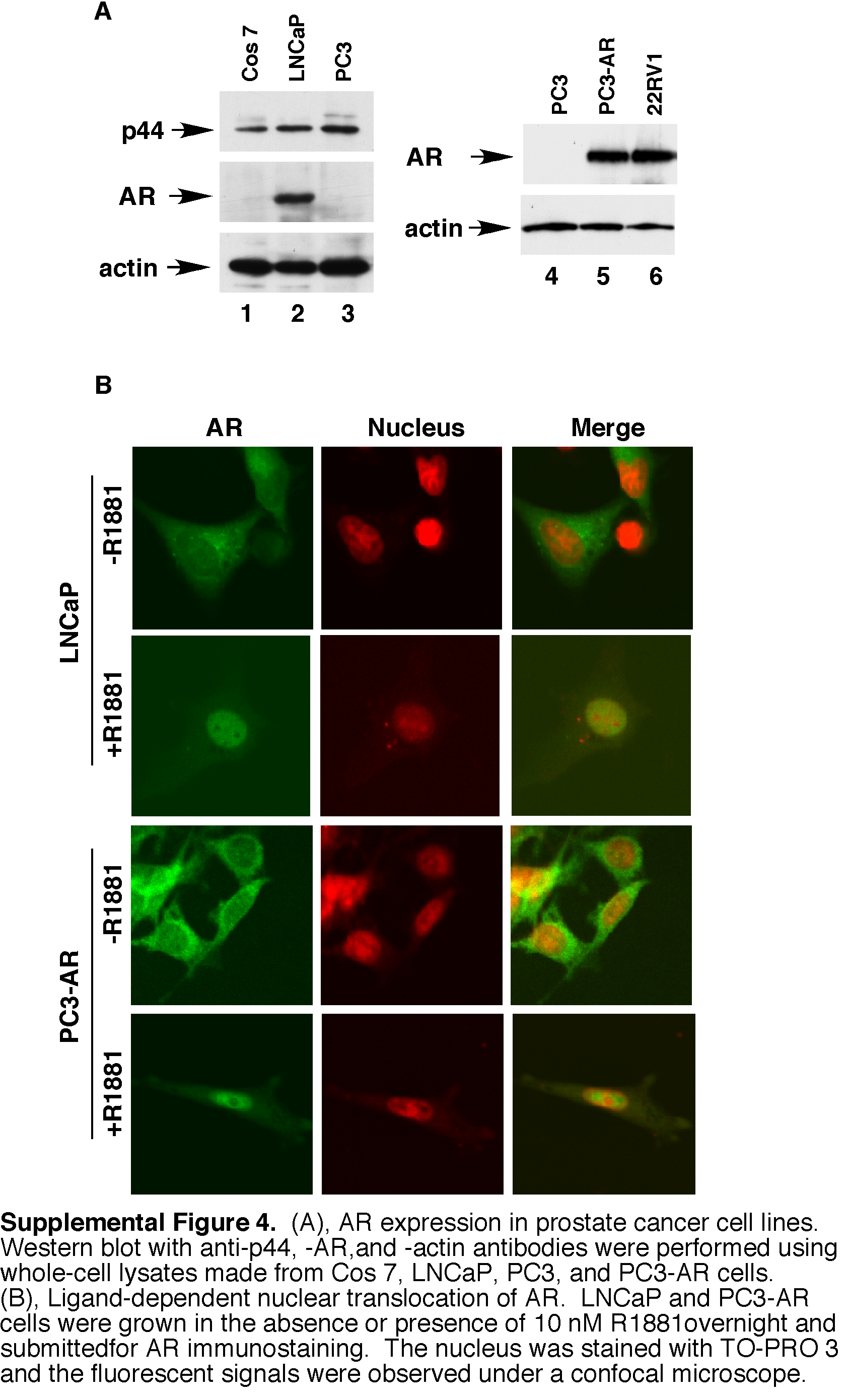

Supplement: Figure S4 — (A), AR expression in prostate cancer cell lines. Western blot with anti-p44, -AR, and -actin antibodies were performed using whole-cell lysates made from Cos 7, LNCaP, PC3, and PC3-AR cells. (B), Ligand-dependent nuclear translocation of AR. LNCaP and PC3-AR cells were grown in the absence or presence of 10 nM R1881overnight and submittedfor AR immunostaining. The nucleus was stained with TO-PRO 3 and the fluorescent signals were observed under a confocal microscope. (TIF) [file pone.0022395.s004.tif]

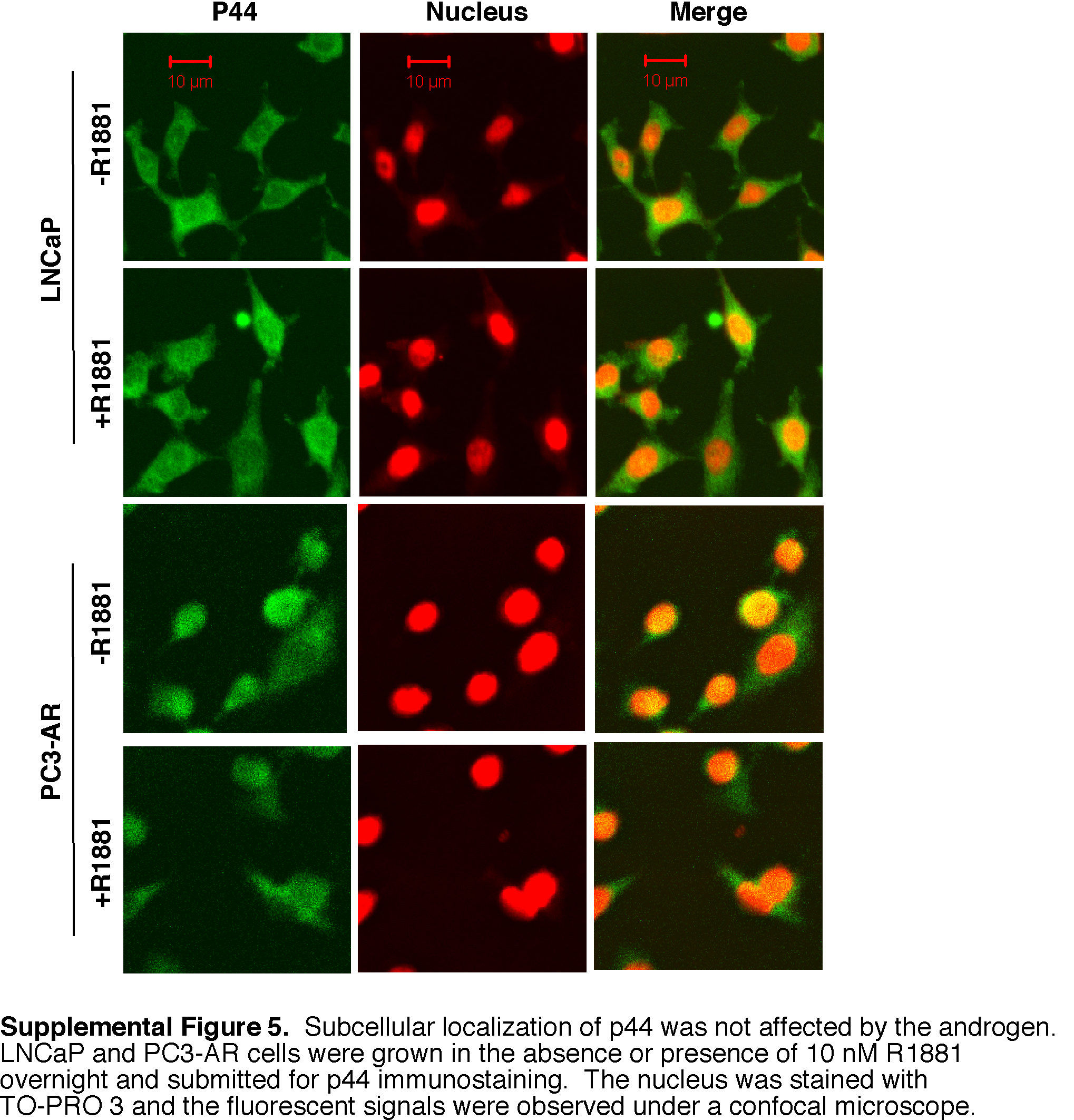

Supplement: Figure S5 — Subcellular localization of p44 was not affected by the androgen. LNCaP and PC3-AR cells were grown in the absence or presence of 10 nM R1881overnight and submitted for p44 immunostaining. The nucleus was stained with TO-PRO 3 and the fluorescent signals were observed under a confocal microscope. (TIF) [file pone.0022395.s005.tif]

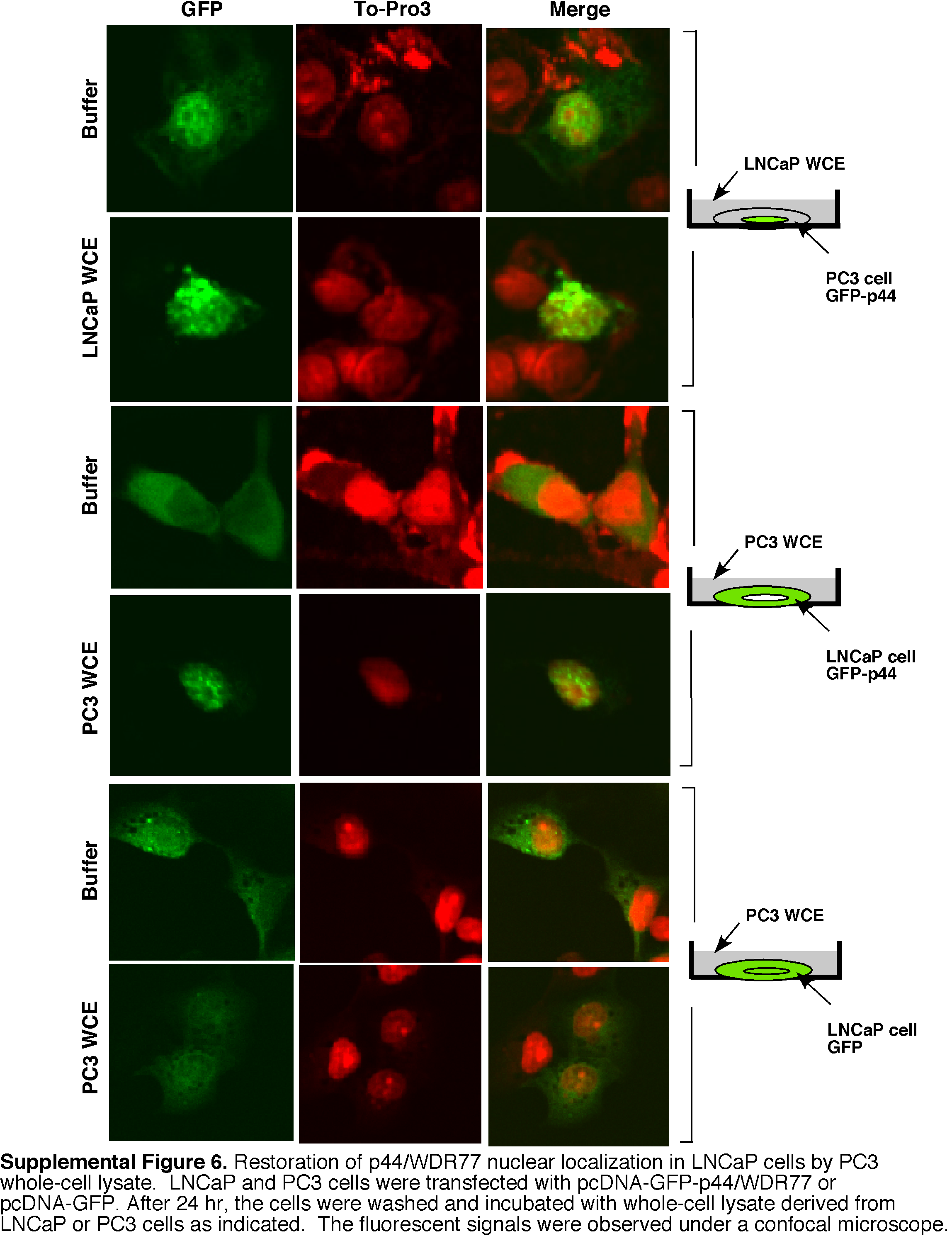

Supplement: Figure S6 — Restoration of p44/WDR77 nuclear localization in LNCaP cells by PC3 whole-cell lysate. LNCaP and PC3 cells were transfected with pcDNA-GFP-p44/WDR77 or pcDNA-GFP. After 24 hr, the cells were washed and incubated with whole-cell lysate derived from LNCaP or PC3 cells as indicated. The fluorescent signals were observed under a confocal microscope. (TIF) [file pone.0022395.s006.tif]

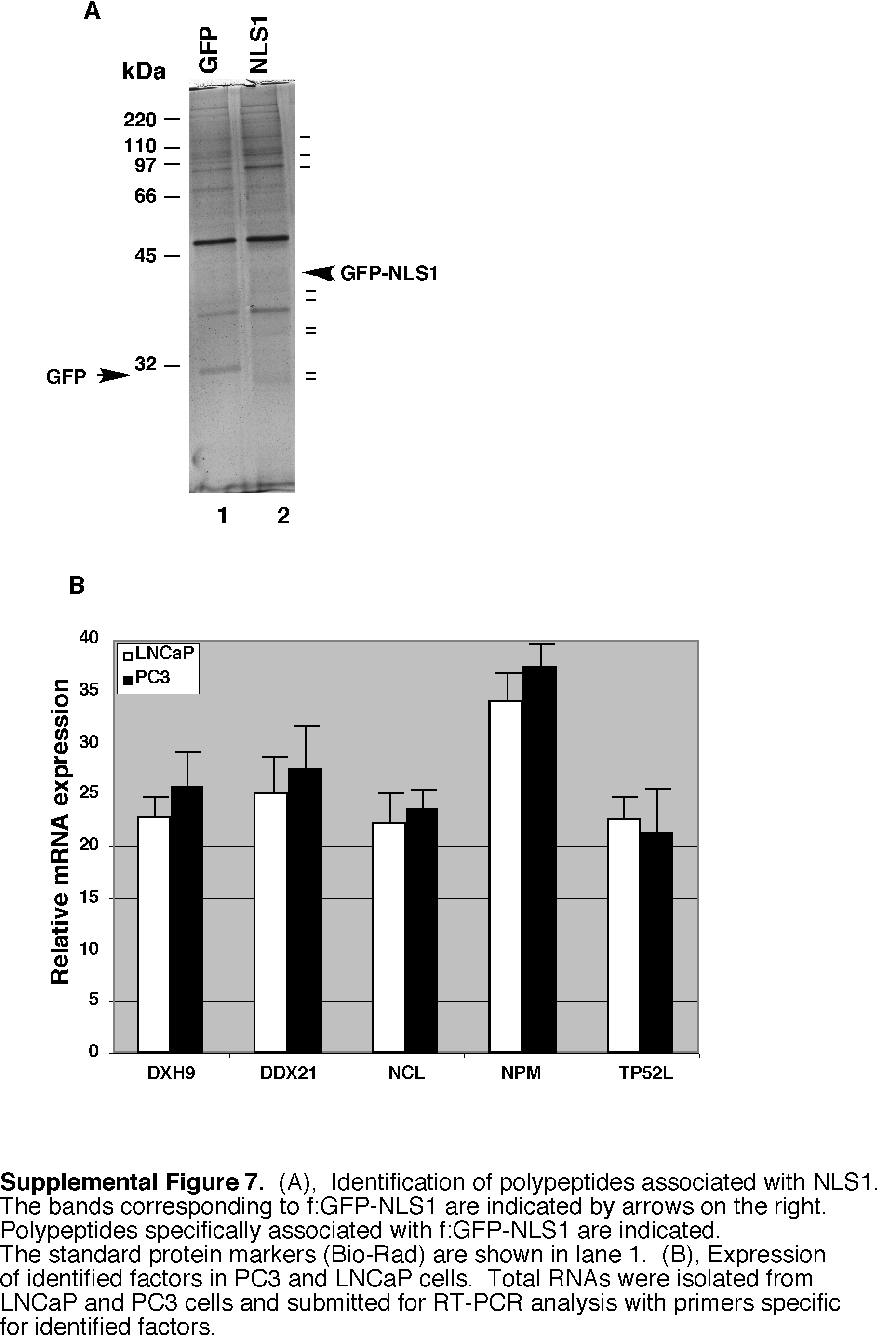

Supplement: Figure S7 — Expression of identified factors in PC3 and LNCaP cells. Total RNAs were isolated from LNCaP and PC3 cells and submitted for RT-PCR analysis with primers specific for identified factors. (TIF) [file pone.0022395.s007.tif]
